# A High Copy Number from a Pharyngeal Swab Is Not Associated with Different Presenting Features in 100 Children with Acute Adenovirus Infection from a Cluster in Italy

**DOI:** 10.3390/children10111788

**Published:** 2023-11-06

**Authors:** Anthea Mariani, Federica Cavallo, Saverio La Bella, Giusi Graziano, Martina Passarelli, Carlo Crescenzi, Daniela Trotta, Maurizio Aricò

**Affiliations:** 1 Pediatrics, S. Spirito Hospital, Azienda Sanitaria Pescara, 65124 Pescara, Italy; anthea.mariani@asl.pe.it (A.M.); federica.cavallo@asl.pe.it (F.C.); daniela.trotta@asl.pe.it (D.T.); 2 Postgraduate School of Pediatrics, University of Chieti-Pescara, 66100 Chieti, Italy; saverio.labella@studenti.unich.it (S.L.B.); martina.passarelli@studenti.unich.it (M.P.); 3 CORESEARCH (Center for Outcomes Research and Clinical Epidemiology), 65122 Pescara, Italy; graziano@coresearch.it; 4 Clinical Microbiology and Virology, S. Spirito Hospital, 65124 Pescara, Italy; carlo.crescenzi@asl.pe.it

**Keywords:** adenovirus, C-reactive protein, copy number

## Abstract

Human mastadenoviruses, frequently denominated adenoviruses (HAdVs), may cause respiratory tract, gastrointestinal or, less frequently, other involvements. Epidemics of HAdV infections occur globally, in communities, and in closed or crowded settings. In our institution, a cluster of infants and children admitted for HAdV infection was recently observed. The aim of this study was to describe the pattern of their presenting features and investigate the possible correlation between the HAdV copy number and the clinical picture. Two main patterns of clinical presentation were observed: 68 patients had mainly respiratory symptoms (pharyngitis n = 67, cough n = 44; tonsillar exudate n = 17; other respiratory signs n = 4) while 26 patients showed prevalent gastrointestinal involvement (diarrhea n = 26, vomiting n = 8). Patients with respiratory symptoms had a significantly higher count of WBC, PMN, and platelets, while CRP level approached statistical significance (*p* = 0.07) for higher values in the patients with diarrhea. In order to explore the impact of selected presenting features, the possible association between the level of CRP and the presence of pharyngeal exudate, cough, vomiting, diarrhea, duration of fever, number of neutrophils, and administration of antibiotics was analyzed. Patients falling in the tertile with more elevated CRP values had tonsillar exudate and diarrhea significantly more often, while those in the lower tertile had a 4.4-day duration fever vs. ≥5.0 days in the remaining patients. Antibiotic therapy was administered more frequently to patients with higher values of CRP (*p* = 0.006). The duration of hospitalization was not associated with the CRP level. The median time from the receipt of a positive HAdV PCR test result to patient discharge was 1 day in 73% of cases. The number of copies of HAdV detected via PCR ranged between 47 million and 15/μL. Falling in the highest tertile of copy number was significantly associated with pharyngitis. The 24 patients with evidence of viral coinfection had no difference in the demographics or presenting features, with the only exception being a significantly higher leukocyte count. The rapid turn-around of the results of the molecular testing of the HAdV genome on a pharyngeal swab allowed us to rapidly diagnose HAdV infection, allowing us to stop antibiotic therapy and immediately discharge the patients, with reduced discomfort for the families and more appropriate use of hospital beds. A high copy number of HAdV from a pharyngeal swab should not be taken as an indicator of worse prognosis, thus allowing for the preferential use of qualitative rather than quantitative assay.

## 1. Introduction 

Human mastadenoviruses, frequently denominated adenoviruses (HAdVs), may cause respiratory tract and gastrointestinal symptoms, while ophthalmologic, genitourinary, and neurologic involvement is far less frequent. HAdV disease is usually self-limiting, although fatal outcome may occur in immunocompromised hosts and occasionally in healthy children and adults [1,2,3,4]. HAdVs have a worldwide distribution and cause 5 to 10 percent of all febrile illnesses in infants and young children throughout the year without seasonality [5]. Serologic evidence of prior adenoviral infection is usually acquired by the age of 10, and by nearly all adults [6]. The transmission of HAdV can occur via aerosol droplets, the fecal–oral route, and through contact with contaminated fomites. Epidemics of HAdV infections occur globally, in communities, and in closed or crowded settings [7,8]. HAdV febrile respiratory illness usually lasts five to seven days, and occasionally up to two weeks. Pharyngitis and coryza are common presentations, but in many cases exudative tonsillitis and cervical adenopathy may be present, mimicking streptococcal infection [9,10]. In a study of 2638 hospitalized children with pneumonia, HAdVs were detected in 15 percent of children younger than five years of age compared with 3 percent of older children [11]. Pneumonia is more severe in infants than in older children, and may be associated with lethargy, diarrhea, and vomiting. Meningoencephalitis, hepatitis, myocarditis, nephritis, neutropenia, and disseminated intravascular coagulation [12,13] may complicate infection. In early 2022, an outbreak of acute hepatitis was identified among young children (most <5 years) in the United Kingdom and Ireland, and other clusters with similar characteristics were subsequently reported in at least 35 countries, including the United States [14,15,16,17,18,19].

We recently observed a cluster of infants and children with HAdV infection. Main presenting features, the number of HAdV copies, the role of CRP for differential diagnosis with bacterial infection, and the assessment of the need for hospitalization and antibiotic therapy were analyzed in a consecutive series of 100 cases.

## 2. Materials and Methods

In this retrospective observational study, we analyzed the clinical records of all children admitted between 1 January 2023 and 18 May 2023. During this time interval, a total of 5080 children aged less than 18 years were seen at our pediatric emergency room, and of them 385 (7.5%) were admitted to our pediatric ward.

The inclusion criteria for the present study were age less than 18 years; in-patient admission between 1 January 2023 and 18 May 2023; and a discharge diagnosis of HAdV infection. Only patients with an unavailable clinical record were excluded.

Viral genome quantification via real-time PCR (q-PCR). The presence of the HAdV genome was investigated on a pharyngeal swab in patients with respiratory or gastro-intestinal symptoms suggesting HAdV infection.

The sample was extracted in about 1 h and 30 min according to a diagnostic protocol that involved the use of the QIAsynphony extraction tool (Qiagen GmbH, Hilden, Germany) and the QIAsynphony DSP Virus/Patogen Kit (Qiagen GmbH, Hilden, Germany). Subsequently, the extracted genetic material was amplified, in about 2 h, according to a genomic amplification protocol (Adenovirus ELITe MGB Kit, Nanogen Advanced Diagnostics, S.p.A. Buttigliera Alta, Torino, Italy), using the CFX96 Real-Time System instrument (Bio-Rad Laboratories Inc., Hercules, CA, USA).

For the quantification of viral DNA copies in different samples, the following formula was used:

$$\mathrm{number}\mathrm{of}\mathrm{copies}=\frac{\mathrm{Ve}\times \mathrm{Quantity}}{\mathrm{Vc}\times \mathrm{Va}\times \mathrm{Ep}}$$

Ve = total volume obtained from extraction (110 µL/Various Materials–165 µL/Blood)
Vc = quantity of sample used in the extraction, 400/µL various materials, 200/µL Blood
Va = eluate volume used in amplification

(a) Blood: 20 µL of eluate + 20 µL of amplification mix
(b) Various materials: 20 µL of eluate + 20 µL of amplification mix

Ep = Procedure Efficiency (100% various materials; 98.95% blood)

The quantitative result was calculated in copies/mL based on a comparison with a straight line created by amplifying four amplification standards of known titer.

Adenovirus antigen detection on stools was performed by using the Meridian Bioscience™ ImmunoCard STAT!™ (Cincinnati, OH, USA), an immunochromatographic assay. Stool swabs were prepared for testing using the ImmunoCardSTAT! Adenovirus assay by adding 700 μL of diluent to a test tube. The swab was placed into the diluent and allowed to soak for 5 min followed by vortexing for 10 s. The swab was then removed from the tube and 150 μL of the remaining solution was tested according to the protocol described below. The assay used immunogold-based technology in a horizontal-flow membrane to detect adenovirus. The stool specimen was diluted 1 to 15 in sample diluent supplied by the manufacturer. The suspension was vortexed and 150 μL was added to the bottom port of the device. The sample mixed with gold particles coated with anti-adenovirus monoclonal antibody and migrated along the nitrocellulose membrane through the capture antibody area and the control (goat anti-mouse antibody) area over a 10 min period at room temperature. After 10 min, the test and control areas were observed for the presence of a blue line across the membrane surface. The control line served as a procedural control to ensure that the sample had migrated the appropriate distance along the membrane. The test line contained anti-adenovirus polyclonal antibody (capture antibody). If adenovirus antigen was present in the sample, a complex formed between the capture antibody and the monoclonal antibody–gold conjugate which could be seen as a blue line in the test area. The absence of a blue line in the test area indicated a negative result.

Clinical data collection. To characterize the pattern of clinical manifestations of HAdV infection, the following fully anonymized data were collected from patient medical charts: age and gender; duration of hospital stay; physical exam findings; leukocyte and neutrophil counts and C-reactive protein (CRP) levels on admission; use of antibiotic therapy. Anonymized data were collected in a specific Excel database.

Statistical analysis. The socio-demographic and clinical characteristics were summarized as mean and standard deviation (SD) and as frequencies and percentages depending on the nature of each variable. The comparison between the two groups of interest (presence/absence of diarrhea, presence/absence of viral coinfection) was made through the *t*-test or Mann–Whitney test in case of continuous variables and through the Chi-squared test or Fisher exact test in case of categorical variables. For the purpose of this study, the patients were also grouped according to CRP tertiles and number of copied tertiles and were formally compared with the ANOVA test or the non-parametric Kruskal–Wallis test and with the Chi-squared test or the Fisher exact test, as appropriate. Statistical significance was reached if *p*-values < 0.05. All the analyses were carried out with SAS software (release 9.4; Cary, NC, USA).

Informed consent for data analysis for scientific purposes was obtained from the parents or legal guardians for all patients. The study was conducted in accordance with the Declaration of Helsinki Ethical Principles and Good Clinical Practices. IRB approval was waived due to study design.

## 3. Results

During the first 4.5 months of the current year 2023, 100 children were hospitalized and then discharged with a diagnosis of HAdV infection. They had a mean age of 4 years, with a preponderance of males. The frequency of individual symptoms is summarized in Table 1.

Evidence of the HAdV genome was obtained via PCR on a pharyngeal swab, with a mean copy number exceeding 30 million/mL. Among 30 patients with some gastrointestinal involvement and thus investigated for HAdV antigen on stools, 6 were positive. Twenty-four patients had evidence of viral co-infection: HHV6 (n = 11), EBV (n = 4), norovirus (n = 4), influenza B (n = 2), rotavirus, RSV, CMV (one each).

Three quarters of the patients received empiric antibiotic therapy. The mean duration of hospitalization was 5 days. None of them required admission to ICU.

Two main patterns of clinical presentation were observed: one group of 68 patients had mainly respiratory symptoms (pharyngitis n = 67; cough n = 44; tonsillar exudate n = 17; other respiratory signs n = 4) and a second group of 26 patients showed prevalent gastrointestinal involvement (diarrhea n = 26; vomiting n = 8) (Table 2).

Furthermore, six additional patients had fever and limited respiratory symptoms but in the presence of another acute, defined clinical condition: urinary tract infection (n = 2), localized skin cellulitis (n = 2), immune thrombocytopenia, neonatal lupus. These six patients with a non-characteristic HAdV infection clinical picture shared low or very low copy numbers at HAdV genome detection via PCR: 752 ± 987 copies/mL.

In order to explore the impact of the selected presenting features, a possible association between the level of CRP and the presence of pharyngeal exudate, cough, vomiting, diarrhea, duration of fever, number of neutrophils, and administration of antibiotics was analyzed (Table 3). Patients falling in the tertile with more elevated CRP values had tonsillar exudate and diarrhea significantly more often, while those in the lower tertile had a 4.4-day duration fever vs. ≥5.0 days in the remaining patients.

Antibiotic therapy was administered more frequently to patients with higher values of CRP (*p* = 0.006). The duration of hospitalization was not associated with the CRP level. The median time from the receipt of positive HAdV PCR test result to patient discharge was 1 day in 73% of cases.

The number of copies of HAdV detected via PCR ranged between 47 million and 15/μL (Table 4). Falling in the highest tertile of copy number was significantly associated with pharyngitis and a non-significant trend to having pharyngeal exudate; patients with higher copy numbers had lower values of gamma-GT, while blood cell count was not affected.

The 24 patients with evidence of viral coinfection had no difference in the demographics or presenting features, with the only exception of a significantly higher leukocyte count. The number of copies of HAdV and ALT values showed a trend to higher values, but those data did not reach statistical significance (Table 5).

None of the study patients developed life-threatening complications, nor required admission to the ICU. All patients were invited, upon discharge, to come back for further evaluation if needed, but none required re-admission for HAdV-infection-related conditions.

## 4. Discussion

On the tail of the annual epidemic of bronchiolitis, during late winter, the rapid spread of HAdV infection was the main reason for the admission of children in our pediatric ward in central Italy. Most HAdV epidemics occur in the winter or early spring, although infections occur throughout the year with no clear seasonality [20]. Although we did not have access to specific epidemiological data in our geographic area for the study period, familial clustering was observed in several cases, suggesting transmission from social exposure to infected individuals in the general population.

Children with persistent fever and upper respiratory symptoms came to the pediatric emergency room for initial evaluation, often after 2–3 days of oral antibiotic therapy. In keeping with the observation made by Jain et al. in their study of 2638 hospitalized children [11], their mean age was of 4 years.

Based on first-level laboratory work-up, children with neutrophilia and higher values of CRP more often received empiric antibiotic therapy, also considering possible invasive bacterial infection. Not surprisingly, fever did not subside, and the option of a higher level of antibiotic coverage was often on the table.

It is interesting to note that the differential diagnosis between HAdV infection and other partially overlapping conditions remains a focus of research. In a recent report, Fabi et al. developed a scoring system based on five clinical parameters and one laboratory parameter to differentiate Kawasaki disease from Multisystem Inflammatory Syndrome in Children (MIS-C) from HAdV infection. By using a multivariable logistic regression analysis, they reported accuracy in recognizing Kawasaki disease from the other overlapping conditions, including 30 cases of HAdV infection. Neutrophilia appears to be the laboratory parameter, which contributes to differentiate HAdV infection from potentially mis-recognized MIS-C [21].

We asked the microbiology lab to start a fast-lane for HAdV PCR testing. Positive results were thus received within 1–2 days in the ward and this was associated with the immediate stop of antibiotic therapy followed by the rapid discharge of children. Reassuring the parents on the identification of the etiology as another case of the ongoing HAdV epidemic allowed them to agree to immediate discharge despite some persisting fever. Overall, this translated into fewer hospital days, fewer antibiotics administered, and a more appropriate use of hospital beds. The expense of a higher number of molecular testing was thus largely compensated even under the mere economical point of view, regardless of the social advantage for the families.

Positive testing was supported by the detail of the copy number of HAdV. How far is this information useful in the clinical practice? In a recent study, Goichman et al. investigated the correlation between HAdV viral load in clinical respiratory samples and respiratory disease severity in pediatric patients. HAdV load in respiratory samples, as measured by Ct values, was found to be negatively correlated with respiratory disease severity in hospitalized patients aged under 9 years [22]. We tried to analyze the possible correlation of copy number with the presenting feature, and we were not able to identify any specific feature predictive for a higher copy number, except for the report of pharyngitis having an inverse relationship with a moderate elevation of gamma-GT. None of the patients with a higher copy number had any severe complication of the clinical course.

The issue of a possible role of the co-infection of HAdV with other viruses has been raised. In a virology surveillance study of 18,603 children seen at seven US sites, HAdV was detected in 1136; of them, 6.1% had co-detection with at least one other respiratory virus (human rhinovirus/enterovirus, respiratory syncytial virus, parainfluenza, influenza, and human meta-pneumovirus), and greater disease severity compared to those with HAdV alone [23]. In a recent study of 16 adenovirus-positive cases from 1 October 2021 to 22 May 2022 in the USA, in parallel adeno-associated virus type 2 (AAV2) was found in blood from 14 cases, compared to 4 (3.5%) of 113 controls (*p* < 0.001) and none of 30 patients with hepatitis of defined etiology. Co-infections of Epstein–Barr virus, human herpesvirus 6, and/or enterovirus A71 were also detected in 12 (85.7%) of 14 cases, with higher herpesvirus detection in cases versus controls (*p* < 0.001). These findings suggest that the severity of the disease is related to co-infections involving AAV2 and one or more helper viruses [24]. In our series, 24 patients had evidence of viral coinfection, in 15/24 cases HHV6 or EBV. Their presenting features were not different from the remaining ones, with the only exception of a higher leukocyte count; yet HAdV copy number and ALT level showed a border-line trend toward higher values in the presence of coinfection. Overall, the disease course in patients with viral coinfection was not more severe.

This study has limitations. In the retrospective analysis, we had no chance to document the number and characteristics of the patients who accessed the emergency room but did not require hospitalization. Thus, the number of patients with HAdV infection was likely higher than that we have obtained through the review of the admitted patients only. Furthermore, we had no chance to obtain any epidemiological information on the real circulation of HAdV in the general population in this time frame. We also did not address the issue of the HAdV genotype in our cluster of children with acute HAdV infection.

## 5. Conclusions

During a cluster of HAdV infection in an urban area, persistent fever drove many families to our pediatric emergency room, and one quarter of admissions to the pediatric ward consisted of patients with HAdV infection. At initial evaluation, neutrophilia and higher values of CRP caused the attending physician to cautiously prescribe empiric antibiotic therapy. In order to explore the impact of selected presenting features, the possible association between the level of CRP and the presence of pharyngeal exudate, cough, vomiting, diarrhea, duration of fever, number of neutrophils, and administration of antibiotics was analyzed. Despite some minor differences, under the clinical point of view, neutrophilia, more elevated CRP values, as well a high copy number of HAdV, did not provide any meaningful hint for treatment tailoring. As a consequence, the preferential use of qualitative rather than quantitative assay for HAdV genome can be allowed. During clusters of acute infectious diseases, bed occupancy may become a relevant issue. The rapid turn-around of the results of the molecular testing of the HAdV genome on a pharyngeal swab led us to rapidly diagnose HAdV infection, allowing us to stop antibiotic therapy and immediately discharge the child, with reduced discomfort for the families and more appropriate use of hospital beds.

## Figures and Tables

**Table 1:** Main features of 100 children with HAdV infection requiring hospital admission.

Variable	N	Mean ± SD or n (%)
*Number*		100
Demographics		
Gender (Female/Male)	100	41/59
Age (years)	100	4.0 ± 3.5
Clinical manifestation		
Pharyngitis	100	87 (87.0)
Cough	100	55 (55.0)
Diarrhea	100	26 (26.0)
Tonsillar exudate	99	25 (25.3)
Vomiting	99	18 (18.2)
Days of fever	70	5.4 ± 2.6
Laboratory		
WBC (cell count/μL)	99	14,690 ± 7985
PMN (cell count/μL)	99	9593 ± 7136
Hb (gr/dL)	100	11.4 ± 1.4
Platelet (cell count × 10^3^/μL)	99	334 ± 134
CRP (mg/L)	99	85 ± 74
ALT (IU/L)	99	44 ± 100
AST (IU/L)	99	49 ± 66
GGT (IU/L)	98	21 ± 42
Fecal antigen	100	
Negative *		24 (24.0)
Positive		6 (6.0)
PCR detection on pharyngeal swab (n. of copies, million/mL)	99	31.1 ± 78.7
Viral co-infection **	100	24 (24)
Treatment		
Antibiotic therapy	100	75 (75.0)
IVIG	100	3 (3.0)
Hospital days	100	5.1 ± 3.4

* 1 case PCR positive, 3 uncertain; ** see text for detail.

**Table 2:** Distribution of presenting features by clinical subgroups.

	Gastrointestinal Symptoms *	Respiratory Symptoms **	*p*-Value ^§^
Number	26	68	
Days of fever	5.5 ± 2.3	5.5 ± 2.7	0.8614
*Laboratory*			
WBC (cell count/μL)	11,160 ± 6316	16,234 ± 8364	0.008
PMN (cell count/μL)	6385 ± 5037	11,046 ± 7610	0.007
Hb (gr/dL)	11.3 ± 1.5	11.4 ± 1.4	0.9563
Platelets (cell count × 10^3^/μL)	290 ± 136	355 ± 128	0.0332
CRP (mg/L)	112 ± 82	80 ± 70	0.0715
ALT (IU/L)	22 ± 17	54 ± 120	0.5702
AST (IU/L)	34 ± 13	57 ± 79	0.2701
GGT (IU/L)	15 ± 13	21 ± 42	0.9723
Antibiotic therapy	17 (65.4)	53 (77.9)	0.2117
Intravenous immunoglobulin	2 (7.7)	1 (1.5)	0.1843
Hospital days	5.2 ± 3.1	5.1 ± 3.6	0.8356

* Diarrhea with or without vomiting. ** Fever, cough, pharyngitis, pharyngeal exudate, no diarrhea. ^§^ *p*-values derived from *t*-test or Mann–Whitney test (for continuous variables) and Chi-squared test or Fisher exact test (for categorical variables).

**Table 3:** Distribution of the presenting features of 100 children with HAdV infection according to CRP tertiles.

	CRP Value (mg/L)			
	0–39	39–105	>105	*p*-Value ^§^
Number	32	33	34	
*Demographics*				
Gender (Female/Male)	14 (43.8)	13 (39.4)	13 (38.2)	0.8917
Age (years)	3.4 ± 3.4	3.7 ± 3.1	4.8 ± 4.1	0.2465
*Clinical manifestation*				
*Tonsillar exudate*	5 (15.6)	5 (15.6)	15 (44.1)	0.0087
*Pharyngitis*	24 (75.0)	30 (93.8)	31 (91.2)	0.067
*Cough*	15 (46.9)	21 (65.6)	18 (52.9)	0.3055
Diarrhea	7 (21.9)	4 (12.5)	15 (44.1)	0.0112
Vomiting	5 (15.6)	4 (12.5)	9 (26.5)	0.3036
Days of fever	4.3 ± 3.2	6.0 ± 2.1	6.0 ± 2.1	0.0627
*Laboratory*				
WBC (cell count/μL)	12,700 ± 7399	16,134 ± 8879	15,176 ± 7542	0.1879
PMN (cell count/μL)	7809 ± 6710	10,559 ± 8038	10,434 ± 6494	0.1495
Hb (gr/dL)	11.8 ± 1.3	11.3 ± 1.5	11.1 ± 1.4	0.1408
Platelets (cell count × 10^3^/μL)	325 ± 127	361 ± 124	310 ± 146	0.2134
ALT (IU/L)	77 ± 147	35 ± 89	22.1 ± 16.4	0.1337
AST (IU/L)	73 ± 101	42 ± 48	35.3 ± 17.6	0.1387
GGT (IU/L)	23 ± 42	26 ± 58	16.4 ± 12.7	0.289
*HAdV diagnostics*				
Fecal antigen	8 (25.0)	7 (21.2)	9 (26.5)	0.8723
Negative *				
Positive	3 (9.4)	1 (3.0)	2 (5.9)	
PCR detection on pharyngeal swab (million of copies/mL)	47,551 ± 117,495	28,283 ± 55,827	19,926 ± 49,220	0.2633
*Treatment*				
Antibiotic therapy	18 (56.3)	27 (81.8)	30 (88.2)	0.0062
Intravenous immunoglobulin	2 (6.3)	0 (0.0)	1 (2.9)	0.3164
Hospital days	4.4 ± 1.9	5.0 ± 2.2	5.3 ± 3.1	0.4904

* 1 case PCR positive, 3 uncertain; ^§^ *p*-values derived from ANOVA or Kruskal–Wallis test (for continuous variables) and Chi-squared test or Fisher exact test (for categorical variables).

**Table 4:** Distribution of the presenting features of 100 children with HAdV infection by tertiles of HAdV copy number/mL.

		Copies			*p*-Value ^§^
		≤954	954–9,490,618	>9,490,618	
Number		32	33	34	
*Demographics*					
Gender	Female	12 (37.5)	12 (36.4)	17 (50.0)	0.4534
	Male	20 (62.5)	21 (63.6)	17 (50.0)	
Age (years)		4.6 ± 4.6	3.5 ± 3.6	3.8 ± 2.2	0.474
*Clinical manifestation*					
Tonsillar exudate		5 (15.6)	7 (21.2)	13 (39.4)	0.0702
Pharyngitis		25 (78.1)	28 (84.8)	34 (100.0)	0.0093
Cough		15 (46.9)	18 (54.5)	22 (64.7)	0.3425
Diarrea		7 (21.9)	9 (27.3)	9 (26.5)	0.8644
Vomiting		5 (15.6)	7 (21.2)	6 (18.2)	0.8439
Days of fever		5.3 ± 3.1	5.1 ± 2.8	5.9 ± 1.9	0.3799
*Laboratory*					
WBC (cell count/μL)		14,781 ± 8508.3	14,935 ± 8260	14,117 ± 7386	0.8512
PMN (cell count/μL)		9409 ± 7822.8	9694 ± 7342	9422 ± 6395	0.8746
Hb		11.5 ± 1.6	11.3 ± 1.4	11.3 ± 1.3	0.6926
Platelets (cell count × 10^3^/μL)		345 ± 161	332 ± 137	329 ± 103	0.9376
ALT (IU/L)		82 ± 162	29 ± 51	24 ± 38	0.0536
AST (IU/L)		75 ± 109	38 ± 24	38 ± 24	0.8209
GGT (IU/L)		30 ± 49	24 ± 54	11 ± 5	0.0002
Fecal antigen	Negative *	8 (25.0)	9 (27.3)	7 (20.6)	0.5752
Antibiotic therapy		24 (75.0)	24 (72.7)	27 (79.4)	0.8097
Intravenous immunoglobulin	No	30 (93.8)	32 (97.0)	34 (100.0)	0.2089
Hospital days		6.2 ± 5.2	5.1 ± 2.4	4.2 ± 1.4	0.1425

* 1 case PCR positive, 3 uncertain; ^§^ *p*-values derived from ANOVA or Kruskal–Wallis test (for continuous variables) and Chi-squared test or Fisher exact test (for categorical variables).

**Table 5:** Distribution of the presenting features of 100 children with HAdV infection according to detection of viral coinfection.

		No Viral Coinfection	Viral Coinfection	*p*-Value ^§^
*Demographics*				
Gender	F	29 (38.2)	12 (50.0)	0.3038
	M	47 (61.8)	12 (50.0)	
Age (years)		3.7 ± 3.3	4.8 ± 4.1	0.1719
*Clinical manifestation*				
Pharyngitis		66 (86.8)	21 (87.5)	1
Cough		45 (59.2)	10 (41.7)	0.132
Diarrhea		22 (28.9)	4 (16.7)	0.2318
Tonsillar exudate		18 (24.0)	7 (29.2)	0.6121
Vomiting		13 (17.3)	5 (20.8)	0.7631
Days of fever		5.4 ± 2.5	5.3 ± 3.1	0.8605
*Laboratory*				
WBC (cell count/μL)		15,528 ± 8263	12,070 ± 6521	0.11
PMN (cell count/μL)		10,590 ± 7191	6477 ± 6102	0.0077
Hb (gr/dL)		11.3 ± 1.4	11.7 ± 1.5	0.2036
Platelet (cell count × 10^3^/μL)		334.7 ± 137.7	333 ± 125	0.8674
ALT (IU/L)		24 ± 35	110 ± 186	0.0877
AST (IU/L)		36 ± 19	94 ± 124	0.1334
GGT (IU/L)		19 ± 36	31 ± 57	0.711
*HAdV diagnostics*				
PCR detection on pharyngeal swab (million of copies/mL)		31,455 ± 72,373	30,242 ± 97,816	0.0926
*Treatment*				
Antibiotic therapy		58 (76.3)	17 (70.8)	0.5887
Hospital days		4.8 ± 2.5	6.2 ± 5.2	0.1173

^§^ *p*-values derived from *t*-test or Mann–Whitney test (for continuous variables) and Chi-squared test or Fisher exact test (for categorical variables). Values in bold are statistically significant.

## Data Availability

No new data were created. Data are contained within the article.
